# Supplementary material for: Conditional chemoconnectomics (cCCTomics) as a strategy for efficient and conditional targeting of chemical transmission
Source: eLife. 2024 Apr 30;12:RP91927. doi: 10.7554/eLife.91927 (PMC11060718; doi:10.7554/eLife.91927)
Supplement: Figure 3—source data 3. [file elife-91927-fig3-data3.zip › Figure 3 source data3 part 2.zip!/Figure 3-Source data 3-part2/1063-1-CRISPResso_Report_az97gqtx.zip!/CRISPResso_on_1063_4/4a.Combined_insertion_deletion_substitution_locations.pdf]

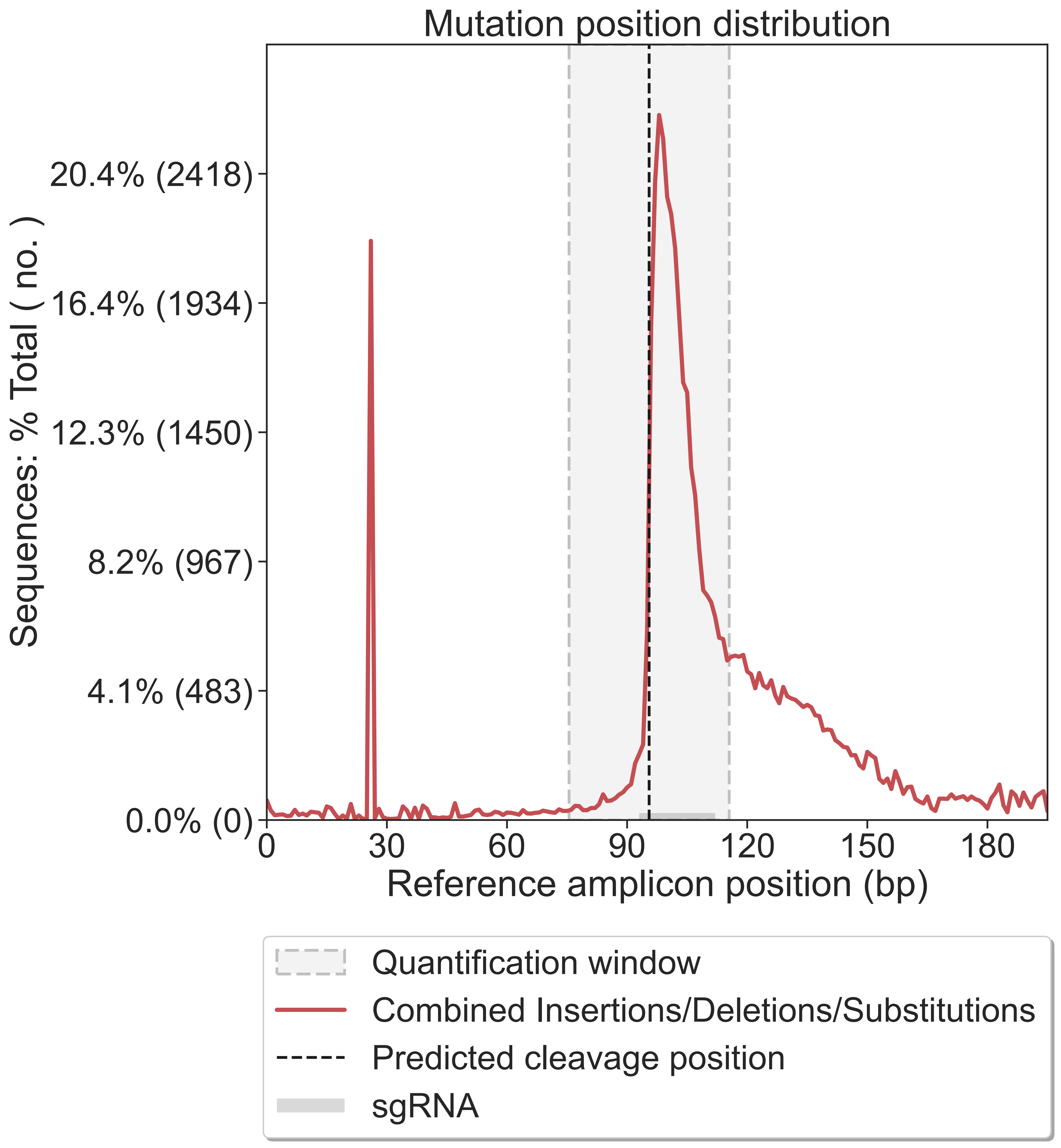
Mutation position distribution
Sequences: % Total (no.)
20.4% (2418)
16.4% (1934)
12.3% (1450)
8.2% (967)
4.1% (483)
0.0% (0)
0
30
60
90
120
150
180
Reference amplicon position (bp)
Quantification window
Combined Insertions/Deletions/Substitutions
Predicted cleavage position
sgRNA
